# STUDENTS GAIN APPRECIATION FOR PATIENT-CENTERED AND TEAM-BASED CARE PARTICIPATING IN A GERIATRIC CASE COMPETITION

**DOI:** 10.1093/geroni/igad104.3604

**Published:** 2023-12-21

**Authors:** Kristine Talley, Marla Berg-Weger, Max Zubatsky, Elaina Osterbur, Rhiannon Williams, Teresa Schicker, Jean Wyman

**Affiliations:** University of Minnesota, Minneapolis, Minnesota, United States; Saint Louis University, St. Louis, Missouri, United States; Saint Louis University, St. Louis, Missouri, United States; Saint Louis University, St. Louis, Missouri, United States; University of Minnesota, Minneapolis, Minnesota, United States; University of Minnesota, Minneapolis, Minnesota, United States; University of Minnesota, Minneapolis, Minnesota, United States

## Abstract

Health science students must attain interprofessional teamwork and collaboration competencies to provide effective, patient-centered care to older adults requiring complex care. However, students typically learn in disciplinary silos and lack engaging opportunities for interprofessional teamwork. To provide students with an interprofessional learning activity the Geriatric Workforce Enhancement Programs (GWEP) at Saint Louis University and the University of Minnesota implemented a Geriatric Case Competition. We present the students’ experience with the 2022 competition. Students assigned to interprofessional teams with a coach had one month to prepare a 20-25 minute presentation of a comprehensive care plan using a simulated complex geriatric patient case. Judges scored team presentations using a rubric of core competencies for interprofessional collaborative practice. Each GWEP held local competitions with winning teams presenting at the inter-GWEP competition held via live videoconferencing. Top scoring teams received prizes. Students completed an eight-item satisfaction survey using a 5 point likert scale (5=poor to 1=excellent). Answers to open ended questions were analyzed qualitatively to find themes about their experience. 132 students joined the competition and 54 completed an evaluation survey (response rate 41%). Students rated their experience as good to excellent with mean ratings on 8 questions ranging from 1.5-2.2 (N=54). Themes included: the importance of patient centered care and the power of multiple perspectives/expertise. Students offered improvements related to student, coach, and judge expectations. In summary, the competition provided a very positive learning activity that helped students value the importance of patient centered care and interprofessional collaboration for older people.

